# Management of analgosedation during noninvasive respiratory support: an expert Delphi consensus document developed by the Italian Society of Anesthesia, Analgesia, Resuscitation and Intensive Care (SIAARTI)

**DOI:** 10.1186/s44158-024-00203-0

**Published:** 2024-09-30

**Authors:** G. Spinazzola, S. Spadaro, G. Ferrone, S. Grasso, S. M. Maggiore, G. Cinnella, L. Cabrini, G. Cammarota, J. G. Maugeri, R. Simonte, N. Patroniti, L. Ball, G. Conti, D. De Luca, A. Cortegiani, A. Giarratano, C. Gregoretti

**Affiliations:** 1 Department of Anesthesia and Intensive Care, Fondazione Policlinico Universitario A. Gemelli IRCCS, Rome, Italy; 2 Catholic University of Sacred Heart, Rome, Italy; 3 Department of Translational Medicine and for Romagna, University of Ferrara, Ferrara, Italy; 4 Department of Emergency and Organ Transplantation (DETO), Section of Anesthesiology and Intensive Care, University of Bari “Aldo Moro’’, Bari, Italy; 5 Department of Anesthesia, Intensive Care and Emergency, SS Annunziata Chieti Hospital, G. D’Annunzio Chieti University Pescara, Pescara, Italy; 6 Department of Anesthesia and Intensive Care of University of Foggia, Foggia, Italy; 7 Department of Biotechnology and Life Sciences, University of Pennsylvania Studies of Insubria, Varese, Italy; 8 Department of Translational Medicine, Università del Piemonte Orientale, Novara, Italy; 9 Anesthesia and Intensive Care Unit, ARNAS Garibaldi Catania, PO “Garibaldi Centro, Catania, Italy; 10 Department of Medicine and Surgery, Università Degli Studi Di Perugia, Perugia, Italy; 11 Anesthesia and Intensive Care San Martino Di Genova, Department of Surgical Sciences and Integrated Diagnosis, University of Genoa, Genoa, Italy; 12 Division of Paediatrics and Neonatal Critical Care, “A. Béclère” Hospital, APHP-Paris Saclay University, Paris, France; 13 Department of Precision Medicine in Area Medical, Surgical and Critical Care. Anesthesia Unit, Resuscitation, and Intensive Care, AOU Policlinico Paolo Giaccone, University of Palermo, Palermo, Italy; 14 Intensive Care Unit, Fondazione G. Giglio, Cefalù, Unicamillus International University, Roma, Cefalù, Italy

**Keywords:** Analgosedation, Respiratory failure, Noninvasive respiratory support, Mechanical ventilation

## Abstract

**Background:**

Discomfort can be the cause of noninvasive respiratory support (NRS) failure in up to 50% of treated patients. Several studies have shown how analgosedation during NRS can reduce the rate of delirium, endotracheal intubation, and hospital length of stay in patients with acute respiratory failure. The purpose of this project was to explore consensus on which medications are currently available as analgosedatives during NRS, which types of patients may benefit from analgosedation while on NRS, and which clinical settings might be appropriate for the implementation of analgosedation during NRS.

**Methods:**

The Italian Society of Anesthesia, Analgesia, Resuscitation and Intensive Care (SIAARTI) selected a panel of experts and asked them to define key aspects of the use of analgesics and sedatives during NRS treatment. The methodology applied is in line with the principles of the modified Delphi and RAND-UCLA methods. The experts developed statements and supportive rationales which were then subjected to blind votes for consensus.

**Results:**

The use of an analgosedation strategy in adult patients with acute respiratory failure of different origins may be useful where there is a need to manage discomfort. This strategy should be considered after careful assessment of other potential factors associated with respiratory failure or inappropriate noninvasive respiratory support settings, which may, in turn, be responsible for NRS failure. Several drugs can be used, each of them specifically targeted to the main component of discomfort to treat. In addition, analgosedation during NRS treatment should always be combined with close cardiorespiratory monitoring in an appropriate clinical setting.

**Conclusions:**

The use of analgosedation during NRS has been studied in several clinical trials. However, its successful application relies on a thorough understanding of the pharmacological aspects of the sedative drugs used, the clinical conditions for which NRS is applied, and a careful selection of the appropriate clinical setting.

**Supplementary Information:**

The online version contains supplementary material available at 10.1186/s44158-024-00203-0.

## Background

Discomfort triggered by agitation, anxiety, pain, delirium, the feeling of dyspnea, and intolerance of the method can be the cause of noninvasive respiratory support (NRS) failure in up to 50% of treated patients [1–3]. Endotracheal intubation and invasive mechanical ventilation as a result of NRS failure remain the main problem, especially in hypoxemic respiratory failure.

While on NRS, the patient’s anxiety, pain, intolerance to unsuitable interfaces, and respiratory fatigue associated with suboptimal patient-ventilator interaction can be managed with an appropriate analgosedation strategy, provided that concomitant optimization of the ventilatory setting and limitation of other factors related to respiratory disease severity are not neglected.

Several studies [2] have shown how the application of an analgosedation strategy during NRS can reduce the rate of delirium, endotracheal intubation, and length of hospital stay in various categories of patients with acute respiratory failure.

However, there are no national or international guidelines defining what analgosedation strategies should be applied in patients with hypoxemic or hypercapnic acute respiratory failure treated with NRS when agitation, anxiety, pain, delirium, sensation of dyspnea, and intolerance might jeopardize the efficacy of ventilatory support.

To fill this gap, a group of intensive care experts selected by the Italian Society of Anesthesia, Analgesia, and Intensive Care (SIAARTI) wrote and approved this good clinical practice document.

The purpose of this document is to evaluate the available drugs and the clinical conditions and settings where analgosedative strategies might be beneficial for patient compliance under NRS, according to the latest scientific literature.

## Methods

### Selection of the expert panel

In January 2023, the Italian Scientific Society of Anesthesia, Analgesia, Resuscitation and Intensive Care (SIAARTI) appointed two coordinators (G. S., C. G.) with clinical and scientific experience to lead a project on noninvasive respiratory support and respiratory failure.

The members of the expert panel were selected by the coordinators. After an initial meeting to discuss the methodology, the different topics were assigned to one or more panel members according to their respective expertise, as follows:

- Evaluate the available evidence.
- Provide supporting statements and justifications in the form of explanatory text.

### Development of research questions

The panel met by videoconference in September 2023 to discuss the project and select topics representing current controversies and open questions, resulting in medicament issues, monitoring issues, clinical setting issues, and respiratory failure-type issues.

In October 2023, consensus on the research questions was assessed anonymously via the SurveyMonkey platform using a Likert rating scale divided into three sections: 1–3 “disagree” or “strongly disagree,” 4–6 “unsure,” and 7–9 “agree.” Agreement (≥ 75% consensus *IQR* 7–9) was achieved for 10 research questions.

The methodological path of the project was outlined by an anesthesiologist intensivist with expertise in methodology (A. C.). It was based on the principles of rapid literature review and the modified Delphi method [4].

### Search strategy and evidence synthesis

In December 2023, the literature review was carried out by two search specialists (J. M., R. S.). The search was conducted on MEDLINE, per the methodology required by SIAARTI. Filters applied included article type (clinical trial, RCT, systematic review, meta-analysis, and review), publication date (within the last 15 years), age (over 18 years, adult), and language (English).

Editorials, letters, case series, case reports, and studies including pediatric patients were excluded for this work.

**Fig. 1:**
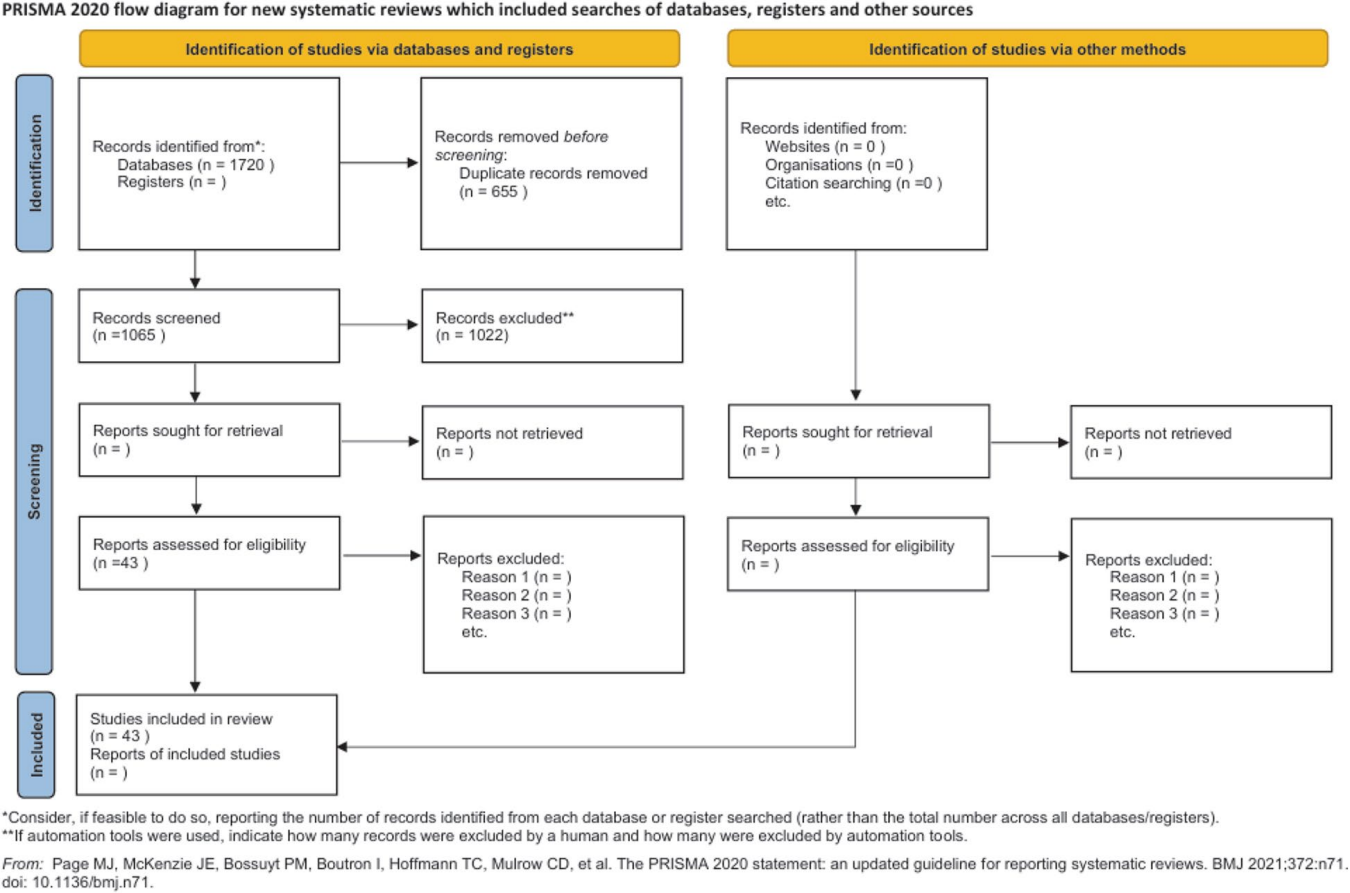
Prisma flow

The search specialists, with the input of the methodologist, created four search strategies by using a combination of keywords and MeSH terms relevant to various clinical questions (Additional File 1). The inclusion/exclusion process was conducted and reported according to PRISMA 2020 [5] (Fig. 1). The list of included papers was then submitted to the panel for review (Additional File 2).

### Formulation of the statements

The panelists were divided into five working groups in January 2024, each with four research questions to develop. Ten statements were formulated between January 2024 and March 2024. The entire panel (with the exclusion of the search specialists and the methodologist) took part in the blind vote. The methodology dictated a maximum of two possible rounds of voting online. The opinion was expressed using an ordinal Likert scale, according to the RAND-UCLA method (minimum score, 1 = completely disagree; maximum score, 9 = completely agree). This scale was divided into 3 sections: 1–3 implied refusal/disagreement (“inappropriate”), 4–6 implied (“uncertainty”), and 7–9 implied agreement/support (“appropriateness”) [6].

A consensus was considered to be reached when as follows:

1. At least 75% of the respondents (excluding the methodologist and the search specialist) assigned a score between 1–3, 4–6, or 7–9, which meant refusal, uncertainty, and agreement with the statement, respectively.
2. The median score was within the same range. The type of consensus was determined by the positioning of the median.

The coordinators reviewed the comments and proposed new wording with eight statements (Table 1), which was approved in a second round of voting. Finally, the draft document was submitted to one external reviewer. The coordinators edited and added to the text as requested.

**Table 1:** Items and statements

**What is the rationale for analgosedation during NRS (NPPV, CPAP, and HFT) treatment and which patients could benefit from it?**	**Statement 1.1** During noninvasive respiratory support (NPPV, CPAP, and HFNT), the use of an analgosedation strategy should be considered in adult patients with hypoxemic or hypercapnic ARF of various etiologies who need to be managed for anxiety, agitation, delirium, dyspnea, and intolerance or pain Regardless of the type of NRS used, close clinical monitoring proportional to the type of support used is mandatory **Statement 1.2** Before the implementation of analgosedation during NRS treatment, the absence of factors specifically related to the condition of respiratory failure (severity or evolution) or inappropriate settings should be carefully assessed to minimize the risk of NRS failure
**What available pharmacological strategies could be implemented for analgosedation during noninvasive respiratory support?**	**Statement 2.1** According to the latest scientific evidence, analgosedation during NRS might be considered to improve adherence to treatments and clinical outcomes. These strategies can be implemented when there are no signs of deterioration, lack of response to NRS, and contraindications to the used pharmacological agents. Although there is no ideal medication and/or protocol for analgosedation during NRS, dexmedetomidine could be considered the drug of choice in patients with closely monitored vital signs (such as blood pressure, heart rate, saturation, and observational sedation scales)
**In the case of analgosedation during noninvasive respiratory support use, how should patients be monitored and what parameters should be considered?**	**Statement 3.1** For analgosedation during NRS, cardiorespiratory monitoring and assessment of consciousness using observational scales should be performed to achieve an appropriate sedation plan and to avoid oversedation. For analgosedation during NRS, cardiorespiratory monitoring and assessment of consciousness using observational scales should be performed to achieve an appropriate sedation plan and to avoid oversedation, monitoring the patients with predefined observational sedation scales and predefined parameters
**What analgosedation targets should be reached according to the reason for NRS use (full treatment or palliative treatment)?**	**Statement 4.1** For analgosedation during NRS, the use of close cardiorespiratory monitoring and assessment of consciousness through observational scales should be performed to achieve an adequate sedation plan and avoid oversedation
**What is the most appropriate timing to start or end analgosedation during NRS?**	**Statement 5.1** The administration of analgesic and/or sedative drugs in patients undergoing NRS can be initiated at two different times: at the start of treatment to improve patient comfort and prevent the onset of patient intolerance or during the NRS as “rescue treatment” at the onset of intolerance and refusal of NRS. However, there are no data in the literature to establish the best time to initiate analgesia e/o sedation during the NRS **Statement 5.2** In the case of NRS intolerance, analgosedation may reduce the incidence of tracheal intubation. However, it should only be used as a last resort after having excluded all the other causes of discomfort and after attempting non-pharmacological measures (such as interface replacement, improving ventilator synchrony, noise reduction, and humidification). Nevertheless, analgosedation should never delay tracheal intubation, potentially masking patient discomfort due to NRS ineffectiveness
**Is the choice of the analgosedative influenced by the type of respiratory failure (acute de novo, chronic exacerbated, postoperative) that led to NRS use?**	**Statement 6.1** The type of respiratory failure and the reasons for prescribing a given drug are among the factors to be considered when choosing analgosedation. In the case of hypercapnic respiratory failure, drugs depressing respiratory activity should be avoided. If intolerance is mainly related to pain, drugs with a predominant analgesic effect should be preferred. In cases of discomfort primarily due to anxiety, drugs capable of producing light sedation or anxiolysis may be more appropriate
**Should analgosedative strategies during NRS be adapted in the case of immunocompromised patients, and are there any specific issues related to this particular population?**	**Statement 7.1** No available data indicates the existence of specificities in analgosedation, in terms of indications, pharmacological techniques, targets, and monitoring needs, for immunocompromised patients. In the absence of specific evidence, the use of analgosedation during NRS should follow strategies used in immunocompetent patients **Statement 7.2** As in immunocompetent patients, it is also crucial for immunocompromised patients to avoid delaying tracheal intubation which remains an urgent and nondeferrable intervention in patients failing NRS
**What are the most appropriate settings to conduct analgosedation during NRS treatment?**	**Statement 8.1** When applying an analgosedation strategy during NRS treatment, it is of pivotal importance to consider the most appropriate monitoring and the clinical setting. For this purpose, the level of intensity of care, the health professional team experience, and the individual patient’s clinical characteristics should be carefully assessed

**Fig. 2:**
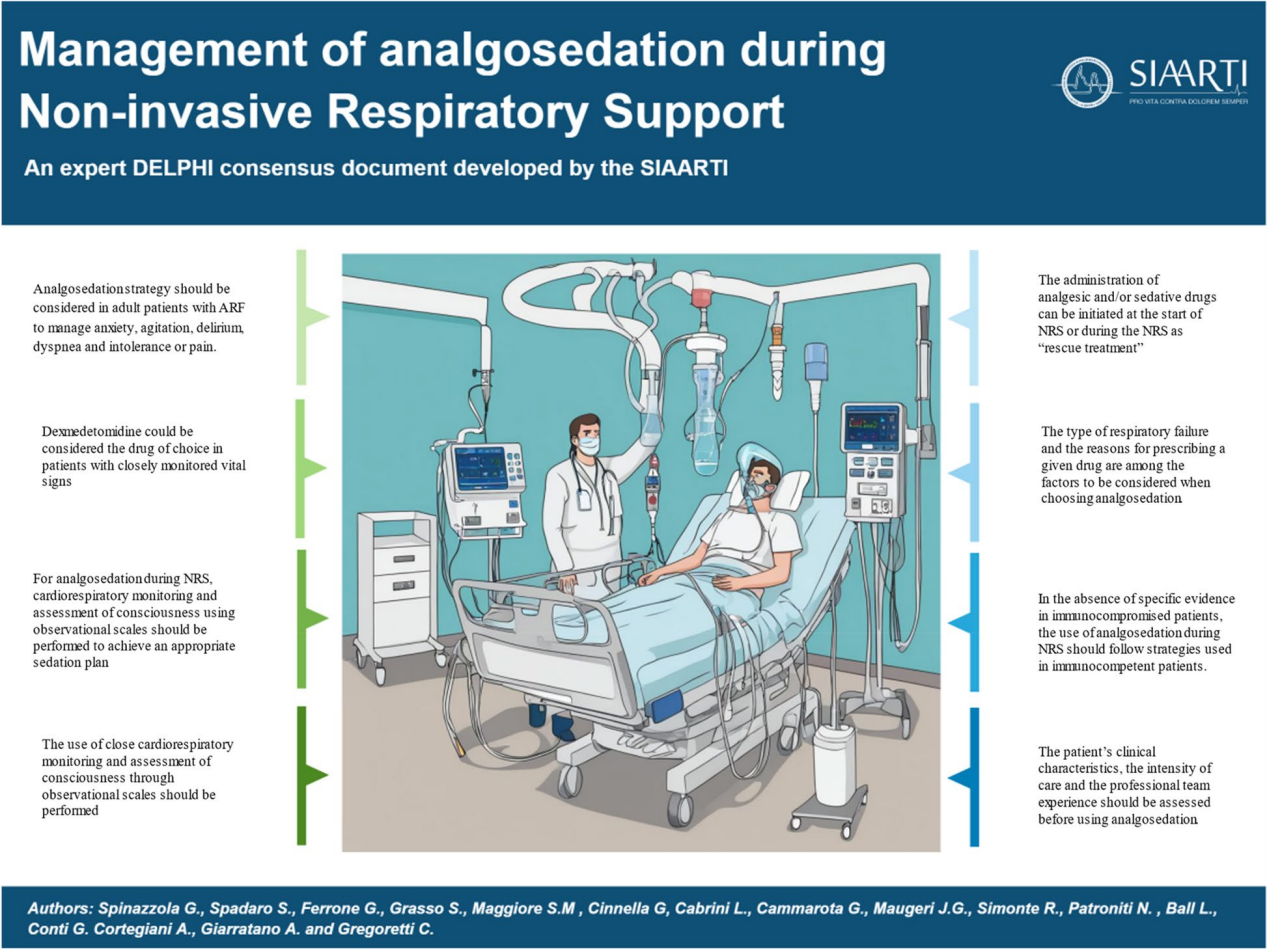
Summary of consensus document

The full version of the Italian document issued by the Italian Society of Anesthesia, Analgesia (Fig. 2), Resuscitation, and Intensive Care (SIAARTI) was published in May 2024 and is freely available on the society’s website in Italian (https://siaarti1934.img.musvc1.net/static/112682/assets/1/BPC%20NIV_SIAARTI2024.pdf).

## Results

### Question 1

What is the rationale for analgosedation during NRS (NPPV, CPAP, and HFNT) treatment, and which patients could benefit from it?

#### Statement 1.1

During noninvasive respiratory support (NPPV, CPAP, and HFNT), the use of an analgosedation strategy should be considered in adult patients with hypoxemic or hypercapnic ARF of various etiologies who need to be managed for anxiety, agitation, delirium, dyspnea, and intolerance or pain.

Regardless of the type of NRS used, close clinical monitoring proportional to the type of support used is mandatory.

#### Statement 1.2

Before the implementation of analgosedation during NRS treatment, the absence of factors specifically related to the condition of respiratory failure (severity or evolution) or inappropriate settings should be carefully assessed, to minimize the risk of NRS failure.

## Rationale

Failure of NRS is the main problem in the management of NRS patients [1–3], because of the need for endotracheal intubation and invasive mechanical ventilation.

NRS is still burdened by a high failure rate (up to 40%, especially in hypoxemic ARF patients) due to agitation, anxiety, pain delirium, dyspnea, and intolerance to respiratory support. Yang et al. in their review and meta-analysis [2] showed how the application of an analgosedation strategy during NRS can reduce the rate of delirium, endotracheal intubation, and hospital length of stay in different categories of patients with ARF. The pharmacological effects of sedatives and/or analgesics used during NRS may explain the results of this study: such drugs have the potential to act on anxiety, dyspnea reduction, and pain perception, which are the main causes of NRS failure. Analgosedation may be able to provide patients with an optimal state of rest and reduction of metabolic stress with subsequent reduction of oxygen consumption and metabolic load on organs.

Poor acceptance of NRS is multifactorial, and the decision to implement analgosedation should be made after evaluating all non-pharmacologic factors (including the evolution of the patient’s ARF and the NRS setting) that may influence the patient’s outcome. Thus, the goal of analgosedation in NRS should include comfort, reduction of dyspnea (in terms of respiratory drive and rate), maintenance of sleep-wake rhythm, homeostasis of metabolism, and attenuation of the immune response to stress, as well as hemodynamic stability [1].

As mentioned above, one category of patients who may benefit from analgosedation in NRS is the dyspneic-anxious patient. In fact, as described by the American Thoracic Society, it is possible to stratify dyspnea based on its neuropathophysiology [7, 8].

Several studies have shown that there is an affective component of dyspnea that can be distinguished from the sensory dimension and may be independently susceptible to manipulation [9–12], especially through the identification and assessment of the anxiety. Therefore, the use of an analgosedation strategy could (and should) be considered in those situations where NRS is clearly indicated and where a careful medical evaluation leads to the identification of anxiety, dyspnea with a strong affective component, or delirium, which might hinder NRS application.

Exhaled tidal volume is one of the parameters that should be closely monitored in hypoxemic patients. It has been shown that during NRS, tidal volumes greater than 9.5 ml/kg of ideal body weight are an index of NRS failure [13]. In this context, analgosedation with drugs that affect drive and/or respiratory rate could be a crucial factor in the optimization of NRS in these patients [14].

In patients with blunt chest trauma, NRS is effective in reducing the rate of intubation and the incidence of pneumonia [14, 15]. However, the absence of dyspnea, control of pain, and control of agitation and anxiety are prerequisites for reducing the incidence of NRS failure. In this context, the use of dexmedetomidine in combination with opioids has proven to be extremely beneficial. Although dexmedetomidine does not affect respiratory drive and timing, it does affect NRS tolerance and reduces respiratory discomfort. This allows for prolonged treatment of trauma patients with NRS. Opioids, also in a multimodal approach with other drug classes (e.g., NSAIDs and paracetamol), are used to control pain [16].

Regardless of adaptation to NRS, it is important to remember that pain should always be treated.

The REDNIVI trial, conducted in postcardiac surgery patients at risk for respiratory failure, compared dexmedetomidine and remifentanil. Dexmedetomidine was highly effective in preventing delirium and improving comfort during NRS; remifentanil was more effective in controlling postoperative pain and reducing respiratory rate. The final effect of the two drugs compared in this particular patient setting was found to be comparable for the improvement of tolerance to the NRS [17]. In conclusion, a pharmacological analgosedative strategy should be considered in patients with hypoxemic and hypercapnic ARF of different origins, who present during NRS use with the abovementioned clinical conditions with the absence of other factors related to the severity of the respiratory disease and/or NRS inappropriate setting, which could be a cause of failure of the method itself.

### Question 2

What available pharmacological strategies could be implemented for analgosedation during noninvasive respiratory support?

#### Statement 2.1

According to the latest scientific evidence, analgosedation during NRS might be considered to improve adherence to treatments and clinical outcomes. These strategies can be implemented when there are no signs of deterioration, lack of response to NRS, and contraindications to the used pharmacological agents. Although there is no ideal medication and/or protocol for analgosedation during NRS, dexmedetomidine could be considered the drug of choice in patients with closely monitored vital signs (such as blood pressure, heart rate, saturation, and observational sedation scales).

## Rationale

Intolerance to NRS is multifactorial [1], and the decision to use sedation should be considered after a careful evaluation of modifiable factors. The choice of the drug to be used should be based on the cause of NRS intolerance. In addition, pharmacological characteristics and clinical effects, as well as the clinical situation and specific needs of each patient, should always be considered [1]. Continuous monitoring by experienced personnel is of pivotal importance, and the doses should be titrated toachieve NRS tolerance while avoiding adverse events.

Furthermore, sedation may cause “intubation delay” especially in “de novo” hypoxemic ARF patients [18].

To analyze the pathophysiological processes that often lead to NRS failure, at least three aspects could be influenced by medications: upper airway patency, respiratory depression, and the affective dimension of dyspnea [1]. In recent years, several authors have demonstrated the efficacy of dexmedetomidine [19], although there is no clear evidence. It reduces the incidence of delirium and tracheal intubation and shortens the length of stay in the ICU and hospital [20, 21].

Dexmedetomidine is a highly selective α2 agonist that exerts its sedative and hypnotic effects by acting on α2 receptors in the locus coeruleus and activating endogenous sleep-promoting pathways, allowing patients to maintain the state of non-REM sleep [22, 23]. The peculiar feature of dexmedetomidine is that it does not lead to respiratory depression and allows the patient to be awakened easily.

In addition, dexmedetomidine has several extremely beneficial effects: anxiolysis, stress reduction, good analgesia, inhibition of salivary secretion, and diuretic effects [23, 24].

Dexmedetomidine also has a pulmonary protective effect by acting on pulmonary vascular contractile mechanisms (by dose dependently modifying catecholamine concentrations such as epinephrine and norepinephrine), pulmonary ischemia-reperfusion injury, and inflammatory factor release [25].

Bradycardia and hypotension have been described as the main drawbacks. However, by avoiding the administration of an initial bolus, these events, especially in the more severe forms, are extremely rare [26].

Propofol is another commonly used drug during NRS. However, special care must be taken with its use because of its potential adverse effects on respiratory drive and upper airway patency. Indeed, a study of electrical activity of the diaphragm (EAdi) showed that propofol had a negative effect on respiratory drive and inspiratory effort [27]. In addition, propofol dose/concentration dependently increases upper airway collapsibility.

Propofol target-controlled infusion (TCI) should be preferred to reduce its adverse effects often associated with infusion accumulation. TCI is a method of administering anesthetic agents based on a pharmacokinetic protocol supported by computerized mathematical calculations of drug concentration. This technique allows for rapid and accurate propofol concentration based on the patient’s clinical response [28]. Unfortunately, TCI cannot be used for prolonged periods for loss of accuracy.

Opioids do not depress respiratory drive, but they do affect respiratory timing. They have sedative and analgesic properties and may be the first choice when the main goal is pain control [29, 30].

In addition, they decrease the perception of dyspnea with a consequent reduction in respiratory rate and improvement in comfort, thus increasing the tolerance of NRS [31]. The major adverse effects associated with opioid infusion include hemodynamic changes and decreased upper airway patency.

Among commercially available opioids, remifentanil has several unique pharmacokinetic characteristics; its metabolism is unaffected by hepatic or renal impairment, and the elimination half-life is less than 10 min, regardless of infusion duration [32]. These properties make remifentanil easy to titrate. It also allows opioid administration with less concern for accumulation and unpredictable and/or delayed recovery. However, to avoid intravenous boluses of the drug, dedicated venous access is needed.

Benzodiazepines, such as midazolam, should be avoided during NRS because of their unpredictable increase in blood concentration. They increase the risk of delirium, cause collapse of the upper airway,and are often associated with respiratory depression [33, 34].

Ketamine usually does not cause respiratory depression at doses used for procedural analgesia or sedation. It decreases airway resistance and hyperreactivity, improves dynamic compliance, and preserves lung volumes while maintaining protective upper airway reflexes [35, 36]. Ketamine may cause hypersalivation, bronchodilation, and delirium. Ketamine should be avoided in patients with decompensated heart failure because of its indirect stimulatory effects on the sympathetic nervous system [37]. The analgosedative effects of low-dose ketamine, especially in the levorotatory formulation, may be useful in patients undergoing NRS with pain and anxiety. However, its administration is too difficult to titrate to avoid its typical adverse effects.

Recent data suggests that less than 20% of patients undergoing noninvasive ventilation receive sedation or analgesia [33]. When medications for analgosedation are used as a single agent, they do not seem to affect the outcomes of failure of noninvasive ventilation or ICU mortality. However, the concomitant use of sedatives and analgesics [33] is associated with an increased risk of noninvasive ventilation failure.

In conclusion, to improve patient tolerance during NRS, pharmacologic sedation can be used, when possible, with a single agent. There is not any perfect drug, and there is not enough data to generalize a preference of one drug over another for any type of patient. Analgosedation can facilitate ventilation, reduce anxiety, promote sleep, and modulate physiologic responses to stress and may therefore represent a viable option to increase the likelihood of success during NRS whenever needed in specific patient and setting.

### Question 3

In the case of analgosedation during noninvasive respiratory support use, how should patients be monitored and what parameters should be considered?

#### Statement 3.1

For analgosedation during NRS, cardiorespiratory monitoring and assessment of consciousness using observational scales should be performed to achieve an appropriate sedation plan and to avoid oversedation, monitoring the patients with predefined observational sedation scales and predefined parameters.

## Rationale

NRS has become increasingly popular in recent decades in the treatment of ARF to reduce the need for endotracheal intubation and its related complications. However, in some patients, NRS fails due to patient agitation or discomfort mainly due to intolerance of the interface (helmet, oro-nasal, and full face masks) [1] but also due to suboptimal patient-ventilator interaction. Initial analyses carried out on intubated and mechanically ventilated patients in pressure support ventilation (PSV) have shown that most asynchronies occur during both the inspiratory and expiratory phases, determined by a delayed or premature cycling of mechanical breaths related to ventilator settings (such as inspiratory trigger, expiratory trigger thresholds, and rapidity of the pressurization ramp) [38].

As described above, one of the parameters to closely monitor during NRS is the expiratory tidal volume. Indeed, tidal volumes greater than 9.5 ml/kg of ideal body weight are an indication of possible NRS failure in hypoxemic ARF [13]. NRS interfaces also have different intrinsic mechanical characteristics. The helmet is associated with a higher incidence of asynchronies when compared with the face mask because the larger internal volume and high compliance of the manufacturing soft material may cause a delay in the inspiratory/expiratory patient’s triggering and/or a slower pressurization rate. Early studies showed that by acting on the parameters regulating inspiratory and expiratory timing, patient-ventilator synchrony may be improved. Costa et al. [38] found that the use of a faster pressurization rate and expiratory flow trigger threshold (i.e., set at a higher peak flow rate) resulted in less inspiratory work and asynchronies, particularly in patients breathing at high respiratory rate. Beyond the optimization of ventilation parameters, adequate patient adaptation during NRS may also require an appropriate analgosedation strategy to optimize patient cooperation, drive, and respiratory timing.

Although there is still no evidence to indicate the best pharmacological strategy, it is generally agreed that it is important to titrate the dosage of drugs until the desired effect is obtained to avoid excessive sedation with possible side effects such as respiratory depression (increased risk of hypercapnia associated with lack of patient respiratory effort, bradypnea, especially in the case of continuous IV sedation [39], hypotension, bradycardia, and especially delirium [7] in the case of benzodiazepine use).

During sedation for NRS, especially if continuous IV drug infusion is used, intensive monitoring becomes mandatory [40], in particular of the following parameters:

- Heart rate, electrocardiogram, and invasive or noninvasive blood pressure (set at least every 15 min max.), and parameters must also be monitored remotely via telemetry.
- Respiratory system dynamics, especially in respiratory rate and possible use of accessory muscles
- Monitoring of ventilator wave curves (pressure and flow)
- Monitoring of measured or estimated expiratory tidal volume in hypoxemic patients (when feasible)
- Pulse oximetry and arterial blood gases measured by arterial sample whenever possible
- Level of consciousness through observational scales such as the RASS (Richmond Agitation Sedation Scale) or the OAA/S (Observer’s Assessment of Alertness/Sedation Scale)
- NRS failure indices according to the used respiratory support (ROX index and HACOR score) [41–43]

Sedation should be thus performed in intensive care-critical care units with dedicated equipment and personnel [40].

### Question 4

What analgosedation targets should be reached according to the reason for NRS use (full treatment or palliative treatment)?

#### Statement 4.1

For analgosedation during NRS, the use of close cardiorespiratory monitoring and assessment of consciousness through observational scales should be performed to achieve an adequate sedation plan and avoid oversedation.

## Rationale

Analgosedation aims to increase the tolerability and comfort of patients during NRS, especially if the interface used is full face or oro-nasal, because these could potentially generate a greater feeling of constriction, suffocation, and therefore agitation than the helmet [1]. The ideal target would be to have a patient alert, conscious, and responsive to verbal stimuli [44]. Drugs that may achieve this aim and minimize side effects are still under study. However, dexmedetomidine [39], remifentanil [32], and propofol pump TCI [44] seem to give the most promising results. Among the various drugs available, one of the most promising is remifentanil because it has an ideal pharmacological profile, given its very short half-life, low accumulation, and organ-independent metabolism. The first results have shown how remifentanil, when used at low dosage, has good sedative effects with minimal or no alteration of the respiratory drive.

At high doses, however, remifentanil may give progressive inhibition of spontaneous respiratory activity up to apnea [30]. Some studies showed an improvement in gas exchanges (decrease in PaCO2 and increase in PaO2/FiO2 ratio) in patients agitated under NRS for ARF [32, 44]; this is probably due to greater patient comfort with less patient-ventilator asynchrony. However, there is still no certainty that analgosedation, however adequate it may be, will increase the success of NRS, because of the paucity of studies specifically addressing sedation [32, 44–46].

NRS is not only used to treat ARF but is also finding space as part of palliative care to improve respiratory symptoms such as wheezing and the sensation of lack of air, very common in end-of-life patients. The role of sedation, in this case, is to achieve symptom control [44–46], as well as improve the tolerability of NRS to increase patient comfort. However, despite international guidelines supporting the discontinuation of any treatment that may unnecessarily prolong patient suffering, there is no consensus on when to suspend NRS, as demonstrated by a recent French study [47], where the opinion of pulmonologists and palliative care specialists on the subject was sought. Pulmonologists consider NRS a life support treatment that, with sedation, can reduce any associated discomfort. Palliativists, however, argue that patient comfort at the end of life has priority compared to respiratory therapy. For this reason, the choice to stop NRS, at the end of life, depends very much on the clinical background but also on ethical, legal, and interpersonal motivations.

### Question 5

What is the most appropriate timing to start or end analgosedation during NRS?

#### Statement 5.1

The administration of analgesic and/or sedative drugs in patients undergoing NRS can be initiated at two different times: at the start of treatment to improve patient comfort and prevent the onset of patient intolerance or during the NRS as “rescue treatment” at the onset of intolerance and refusal of NRS. However, there are no data in the literature to establish the best time to initiate analgesia e/o sedation during the NRS.

#### Statement 5.2

In the case of NRS intolerance, analgosedation may reduce the incidence of tracheal intubation. However, it should only be used as a last resort after having excluded all the other causes of discomfort and after attempting non-pharmacological measures (such as interface replacement, improving ventilator synchrony, noise reduction, and humidification). Nevertheless, analgosedation should never delay tracheal intubation, potentially masking patient discomfort due to NRS ineffectiveness.

#### Statement 5.3

Analgosedation should be continued as long as the reasons for which it was prescribed persist or as long as no related adverse events (respiratory, hemodynamic, or other) occur. Nevertheless, analgosedation should be always titrated using validated scores. The most commonly used scores in the ICU are the Ramsay Sedation Scale, Richmond Agitation Sedation Scale (RASS), and Riker Sedation Agitation Scale (SAS). The optimal suggested sedation level during NRS corresponds to a patient who is calm, cooperative, and easily awakened (Ramsay scales 2–3, RASS-1, SAS 3–4). The level of sedation should be measured at regular time points throughout NRS (Table 2).

**Table 2:** Sedation scores in ICU

**Ramsay Sedation Scale**	
Clinical score	Patient characteristics
1	Awake; agitated or restless or both
2	Awake; cooperative, oriented, and calm
3	Awake; only responding to commands
4	Sleepy; immediate response to mild glabellar stimulation or a strong auditory stimulus
5	5 Asleep; slow response to light glabellar tapping or strong auditory stimulus
6	Asleep; no response to glabellar stimulation or strong auditory stimuli
**Richmond Agitation Sedation Scale (RASS)**	
Clinical score	Patient characteristics
+4	Combative
+3	Very agitated
+2	Agitated
+1	Restless
0	Awake and quiet
−1	Sleepy
−2	Slightly sedated
−3	Moderately sedated
−4	Deeply sedated
−5	Unarousable
**Riker Sedation Agitation Scale (SAS)**	
Clinical score	Patient characteristics
7	Dangerous agitation
6	Very agitated
5	Agitated
4	Calm and cooperative
3	Sedated
2	Very sedated
1	Inexcitable

## Rationale

A high number of patients can present discomfort, claustrophobia, intolerance to the interface, increased effort, and other complaints, with an incidence of up to 50% [46]. The initiation of analgosedation may be considered if such complaints arise and hinder the continuation of treatment: this is the most investigated indication in the literature [1, 2, 16, 33, 44, 45, 47–53]. The degree of patient discomfort can be assessed through an intolerance score (1 to 4 points) [54], which evaluates patient compliance to the treatment (1 optimal tolerance to 4 worst tolerance with attempt to tear the interface off).

Analgosedation may also be initiated as early as the start of the NRS, to try to reduce the incidence of these complications and promote treatment tolerance in specific types of patients such as post-cardiac surgery [55], patients with thoracic trauma [14], and/or for longer periods, especially under special conditions such as prone position in Covid 19 [56, 57]; in a single study, the use of sedation was intended to promote sleep, whose NRS-related fragmentation may be a cause of treatment refusal [58].

However, analgesics and/or sedatives should be initiated when it is no longer been possible to improve the patient’s tolerance to respiratory support by removing the causes of discomfort: in particular, this may require replacement or rotation of different interfaces, optimized humidification, noise reduction, reduction of air leakage, modification of respiratory parameters, improvement of patient-ventilator synchrony, patient motivation, and other similar measures [1, 39, 49]. More importantly, it should be remembered that patient discomfort may be due to the ineffectiveness of NRS in treating the underlying ARF: in such circumstances, when there is a worsening of arterial blood gases coupled with an increase in respiratory rate/effort and interface intolerance, the correct intervention consists (if considered appropriate for the specific patient) of tracheal intubation and subsequent invasive ventilation. This decision should never be delayed by a pharmacological approach in a dyspneic patient [1, 39, 48].

The systematic review carried out for the present good clinical practices did not identify any clinical studies or other forms of publication (expert opinions or narrative reviews) that addressed the continuation and discontinuation of analgosedation during NRS. In analogy to the most recent guidelines on sedation [54], it seems reasonable to continue analgosedation as long as the reasons for prescribing it persist or as long as no adverse events (respiratory or otherwise) related to it occur.

In a recent meta-analysis based on 14 RCTs [2], the use of analgosedation was found to reduce the incidence of tracheal intubation in patients undergoing NRS. However, analgosedation should be titrated, using validated scores, considering the favorable or unfavorable evolution of respiratory failure and NRS treatment.

Abundant data in the literature indicate that structured sedation and agitation assessment scores are still underused, despite their routine use being strongly recommended by the guideline on sedation in intensive care. The rationale is that the scores allow for uniform stratification of patients, standardized application of sedation protocols, and titration of sedative and analgesic medication dosing over time. The most commonly used scores in the ICU are the Ramsay Sedation Scale, Richmond Agitation Sedation Scale (RASS), and Riker Sedation Agitation Scale (SAS) (see Table 2). The optimal level of sedation suggested during NRS is that which corresponds to a calm, cooperative, and easily awakened patient (Ramsay scale 2 to 3, RASS 0 to 1, SAS 3 to 4). The level of sedation should be measured at regular time points throughout NRS.

### Question 6

Is the choice of the analgosedative influenced by the type of respiratory failure (acute de novo, chronic exacerbated, postoperative) that led to NRS use?

#### Statement 6.1

The type of respiratory failure and the reasons for prescribing a given drug are among the factors to be considered when choosing analgosedation. In the case of hypercapnic respiratory failure, drugs depressing respiratory activity should be avoided. If intolerance is mainly related to pain, drugs with a predominant analgesic effect should be preferred. In cases of discomfort primarily due to anxiety, drugs capable of producing light sedation or anxiolysis may be more appropriate.

## Rationale

Although there are no studies specifically addressing this aspect, the type of respiratory failure and the reasons for prescribing NRS are certainly factors to consider carefully when choosing the type of analgosedation, taking the properties of different categories of drugs and their positive and negative effects into account. Most published studies focus on patients with COPD and hypoxemic respiratory failure with a PaO_2_/FiO_2_ ratio > 150. Limited but encouraging data is available on the use of analgosedation to facilitate the sleep-wake cycle, predominantly in cardiological patients. Specifically, in cases of hypercapnic respiratory failure, drugs that depress respiratory activity should ideally be avoided; if intolerance is primarily related to pain (either due to the interface or postoperative pain possibly exacerbated by NRS), drugs with a predominant analgesic effect should be preferred; in cases of discomfort due to anxiety or nonspecific causes, drugs capable of producing light sedation may be more appropriate.

### Question 7

Should analgosedative strategies during NRS be adapted in the case of immunocompromised patients, and are there any specific issues related to this particular population?

#### Statement 7.1

No available data indicates the existence of specificities in analgosedation, in terms of indications, pharmacological techniques, targets, and monitoring needs, for immunocompromised patients. In the absence of specific evidence, the use of analgosedation during NRS should follow strategies used in immunocompetent patients.

#### Statement 7.2

As in immunocompetent patients, it is also crucial for immunocompromised patients to avoid delaying tracheal intubation which remains an urgent and nondeferrable intervention in patients failing NRS.

## Rationale

There are no studies specifically investigating the use of analgosedation in immunocompromised patients. Moreover, randomized controlled trials and observational studies on the use of NRS in the immunocompromised patient population do not report specific indications for the use of analgosedation in treatment protocols, nor do they provide data on the incidence of recourse to analgosedation among the outcomes [16, 59–61]. Consequently, meta-analyses that have examined the use of analgosedation in NRS do not include immunocompromised patients [2, 62]. In the absence of specific data, the information available from the literature on the use of analgosedation in noninvasive respiratory support for immunocompetent patients is also applicable to immunocompromised patients.

The initial randomized controlled trials in the early 2000s [16, 59] showed a reduction in mortality in immunocompromised patients for whom the use of NRS could avoid intubation. However, the mortality in immunocompromised patients requiring intubation has decreased over the years [61–64], and more recent randomized trials have failed to demonstrate the effectiveness of NRS in reducing mortality in immunocompromised patients [61–64]. Furthermore, several studies have shown an increase in mortality associated with delayed intubation [65–69].

For these reasons, analgosedative drugs should be used with caution in immunocompromised patients, and delayed intubation should be avoided due to the risks associated with endotracheal intubation.

### Question 8

What are the most appropriate settings to conduct analgosedation during NRS treatment?

#### Statement 8.1

When applying an analgosedation strategy during NRS treatment, it is of pivotal importance to consider the most appropriate monitoring and the clinical setting. For this purpose, the level of intensity of care, the health professional team experience, and the individual patient’s clinical characteristics should be carefully assessed.

## Rationale

NRS is increasingly used outside the intensive care unit (ICU) for managing the early stages of acute and exacerbated chronic respiratory failure [70–73]. Especially when administered over a prolonged period, NRS requires optimization of patient comfort through non-pharmacological measures, often combined with analgosedation [70]. The pharmacological approach may involve intermittent or continuous administration of analgesics and/or sedatives depending on the properties of the molecules used [70]. Analgosedation is commonly reserved for application in ICUs because it ensures continuous monitoring of vital parameters and prompt intervention in managing emergencies during treatment. However, experiences of analgosedation outside ICUs have been reported during NRS, particularly in sub-intensive care units (SICUs) [39, 53] in emergency departments [73] and in postanesthesia recovery rooms [55], as well as in general wards [39]. Specifically in general wards, an intermittent analgosedation regimen during NRS was applied in 21% of patients without do-not-intubate (DNI) orders and in 41% of DNI patients [39]. Analgosedation with continuous IV infusion of medications was administered in 18% of patients without DNI and 41% of DNI subjects [39]. Higher rates of analgosedation were recorded in the emergency department, exceeding 50% of cases regardless of DNI status [61]. Intermittent analgosedation regimens were based on the administration of risperidone and haloperidol, primarily [39]. Conversely, dexmedetomidine was the most used drug for continuous infusion analgosedation [39]. It is important to note that in the retrospective study [39], general wards were transformed into intensive care environments with 24-h monitoring, managed by experienced respiratory therapy staff. Dexmedetomidine has proven effective in improving patient comfort during NRS in respiratory SICUs [53], in emergency departments [55], and postanesthesia recovery rooms for thoracic surgery [55]. Specifically, a dexmedetomidine analgosedation regimen has been associated with reduced intubation rates and delirium, as well as shortened duration of NRS and ICU stays [2], although increased incidence of sinus bradycardia and hypotension has been reported [2, 55]. Consequently, continuous monitoring of vital signs and clinical conditions of patients undergoing NRS and analgosedation is mandatory [71]. Therefore, although the administration of sedative and/or analgesic drugs is generally associated with reduced intubation rates and delirium incidence [66], consideration must be given to the pharmacokinetic and pharmacodynamic profiles of individual drugs used, according to the patient type requiring NRS and analgosedation [71].

In conclusion, the choice of an analgosedation regimen should be based on the intensity of care and monitoring, staff experience in managing pharmacological regimens, recognizing/managing emergencies during treatment, and patient clinical characteristics.

## Conclusions

In conclusion, analgosedation should be carefully considered in patients with discomfort of various origins during NRS treatment. Its application should be evaluated after excluding other causes of discomfort, mainly related to the clinical progression of the underlying respiratory disease and/or incorrect NRS setting. Analgosedatives should be targeted to the patient’s respiratory disease and the cause of patient discomfort (e.g., anxiety, agitation, delirium, dyspnea, and tachypnea). Finally, these strategies require careful cardiorespiratory monitoring in an appropriate clinical setting.

## Supplementary Information

Supplementary Material 1. Search Strings.

Supplementary Material 2. List of included papers.

## Data Availability

No datasets were generated or analysed during the current study.

## Abbreviations

NRS: Noninvasive respiratory support
HFNT: High-flow nasal therapy
CPAP: Continuous positive airway pressure
NPPV: Noninvasive positive pressure ventilation
SIAARTI: Italian Society of Anesthesia, Analgesia, and Intensive Care
ICU: Intensive care unit
SICU: Sub-intensive care unit
RASS: Richmond Agitation Sedation Scale
SAS: Riker Sedation Agitation Scale
DNI: Do-not intubate
COPD: Chronic obstructive pulmonary disease
TCI: Target-controlled infusion
EAdi: Electrical activity of the diaphragm
ARF: Acute respiratory failure
IV: Intravenous
