# Supplementary material for: Management of analgosedation during noninvasive respiratory support: an expert Delphi consensus document developed by the Italian Society of Anesthesia, Analgesia, Resuscitation and Intensive Care (SIAARTI)
Source: J Anesth Analg Crit Care. 2024 Sep 30;4:68. doi: 10.1186/s44158-024-00203-0 (PMC11441104; doi:10.1186/s44158-024-00203-0)
Supplement: Supplementary file 1 — Supplementary Material 1. Search Strings. [file 44158_2024_203_MOESM1_ESM.docx]

***Additional File 1: Search Strings***

**- What is meant by analgosedation?**

**- What is the rationale for the use of analgosedation in adult patients undergoing noninvasive respiratory support (NPPV, CPAP, and HFNT)?**

**- What are the pharmacological strategies available for analgosedation during noninvasive respiratory support?**

**- What parameters should be monitored and what monitoring should be used during analgesia in patients on noninvasive respiratory support?**

(((analgosedation) OR (sedation) OR (analgo-sedation) OR (Hypnotic) OR (Sedative) OR (Analgesic) OR (Propofol) OR (Fentanyl) OR (Remifentanil) OR (Dexmedetomidine) OR (Haloperidol) OR (Midazolam)) AND ((non invasive ventilation) OR (non invasive respiratory support) OR (NIV) OR (non-invasive positive pressure ventilation) OR (CPAP) OR (HFNT) OR (HFNO) OR (high-flow nasal therapy) OR (high-flow nasal oxygen) OR (high-flow nasal cannula) OR (HFNC) OR (continuous positive airway pressure) OR (Non invasive positive pressure ventilation) OR (NPPV))) AND (((“Monitoring Parameters” OR “Monitoring Strategies” OR “Patient Assessment” OR “Safety Measures” OR “Monitoring”)) OR ((Rationale) OR (Justification) OR (Reasons) OR (Purpose)))

**RESULTS: 439**

**-What are the targets of analgosedation according to the goals of noninvasive respiratory support (full treatment or palliative treatment)?**

**- When to start analgosedation in patients undergoing noninvasive respiratory support? How and when terminate it?**

((analgosedation) OR (sedation) OR (analgo-sedation) OR (Hypnotic) OR (Sedative) OR (Analgesic) OR (Propofol) OR (Fentanyl) OR (Remifentanil) OR (Dexmedetomidine) OR (Haloperidol) OR (Midazolam)) AND ((non invasive ventilation) OR (non invasive respiratory support) OR (NIV) OR (non-invasive positive pressure ventilation) OR (CPAP) OR (HFNT) OR (HFNO) OR (high-flow nasal therapy) OR (high-flow nasal oxygen) OR (high-flow nasal cannula) OR (HFNC) OR (continuous positive airway pressure) OR (Non invasive positive pressure ventilation) OR (NPPV))) AND (((Treatment Outcome) OR (Palliative Care) OR (Full Treatment)) OR ((Timing) OR (Initiation) OR (Termination) OR (Discontinuation)))

**RESULTS: 287**

**-What type of patient needs analgosedation during noninvasive respiratory support?**

**- Can the choice of the type of analgosedation be influenced by the type of respiratory failure (acute de novo, chronic exacerbated, postoperative) that required the use of noninvasive respiratory support?**

**- Are there specificities of analgosedation in immunocompromised patients?**

(((analgosedation) OR (sedation) OR (analgo-sedation) OR (Hypnotic) OR (Sedative) OR (Analgesic) OR (Propofol) OR (Fentanyl) OR (Remifentanil) OR (Dexmedetomidine) OR (Haloperidol) OR (Midazolam)) AND ((non invasive ventilation) OR (non invasive respiratory support) OR (NIV) OR (non-invasive positive pressure ventilation) OR (CPAP) OR (HFNT) OR (HFNO) OR (high-flow nasal therapy) OR (high-flow nasal oxygen) OR (high-flow nasal cannula) OR (HFNC) OR (continuous positive airway pressure) OR (Non invasive positive pressure ventilation) OR (NPPV))) AND (((Patient Selection [Mesh]) OR (Indications) OR ((Respiratory Insufficiency[Mesh]) OR (Acute Respiratory failure) OR (Chronic Respiratory failure) OR (COPD) OR (Immunocompromised) OR (Immunosuppression) OR (Immunodeficiency)))

**RESULTS: 44**

**- In what setting (intensivist and/or nonintensivist) is it advisable to perform analgosedation during noninvasive respiratory support?**

(((analgosedation) OR (sedation) OR (analgo-sedation) OR (Hypnotic) OR (Sedative) OR (Analgesic) OR (Propofol) OR (Fentanyl) OR (Remifentanil) OR (Dexmedetomidine) OR (Haloperidol) OR (Midazolam)) AND ((non invasive ventilation) OR (non invasive respiratory support) OR (NIV) OR (non-invasive positive pressure ventilation) OR (CPAP) OR (HFNT) OR (HFNO) OR (high-flow nasal therapy) OR (high-flow nasal oxygen) OR (high-flow nasal cannula) OR (HFNC) OR (continuous positive airway pressure) OR (Non invasive positive pressure ventilation) OR (NPPV))) AND (((Intensive care unit) OR (ICU) OR (Clinical setting) OR (General ward) OR (Ambulatory care)))

**RESULTS: 553**
